# Validation of immunoassays for the *Chlamydia trachomatis* antigen Pgp3 using a chimeric monoclonal antibody

**DOI:** 10.1038/s41598-023-33834-4

**Published:** 2023-05-04

**Authors:** Brook Goodhew, Xiaoling Tang, Jason Goldstein, Joo Lee, Diana Martin, Sarah Gwyn

**Affiliations:** 1 Division of Parasitic Diseases and Malaria, Centers for Disease Control and Prevention, Atlanta, Georgia; 2 Division of Scientific Resources, Centers for Disease Control and Prevention, Atlanta, Georgia

**Keywords:** Biological techniques, Immunology

## Abstract

Seroepidemiology, or measuring antibodies to pathogens to estimate population-level exposure, can provide useful public health data. The tests used, however, often lack sufficient validation data due to absence of a gold standard. For many pathogens, serum antibodies can be detected long after resolution of infection, but infection status is often used as a gold standard for antibody positivity. To ensure that recently developed antibody tests for seroepidemiology of *Chlamydia trachomatis* (Ct), the causative agent of urogenital chlamydia and the blinding eye disease trachoma, have high performance, we generated a chimeric antibody to the immunodominant Ct antigen Pgp3. Two clones were selected to evaluate the test performance of three assays to measure antibodies to Pgp3: multiplex bead assay (MBA), enzyme-linked immunosorbent assay (ELISA), and lateral flow assay (LFA). Overall, each assay demonstrated high accuracy and precision when tested using either clone, and the clones were stable when stored at − 20 °C and 4 °C for almost 2 years. The limit of detection was similar for MBA and LFA, but almost a log-fold higher (i.e. less sensitive) using ELISA. Overall, the chimeric antibodies represent stable control reagents for tests with robust performance and will facilitate deployment of these tests to other laboratories.

## Introduction

Seroepidemiology of infectious agents can estimate population-level exposure to infections. There is increasing interest in seroepidemiology to understand the transmission of *Chlamydia trachomatis*^1,2^, the etiologic agent for both the sexually transmitted chlamydial infections and the blinding eye disease trachoma. Trachoma is a neglected tropical disease (NTD) with a target of elimination as a public health problem by 2030. Repeated infections with ocular strains of *C. trachomatis* can lead to scarring of the upper tarsal conjunctiva, which can cause in-turning of the lashes to rub against the eye, that can then cause corneal opacity and blindness. The programmatic target related to infection is the clinical sign trachomatous inflammation—follicular (TF) in 1–9-year-olds living in affected areas and addressed through antibiotic distribution of azithromycin and hygiene interventions to control spread of infection. Once the district-wide target of < 5% TF is met and interventions are removed, there is the possibility of recrudescence and post-elimination surveillance will need to be undertaken. Tests for antibodies against the *C. trachomatis* antigen Pgp3 to support trachoma programs have been developed for multiplex bead assay (MBA)^3^, enzyme-linked immunosorbent assays (ELISA)^4^, and lateral flow assay (LFA)^5,6^ and have been evaluated in population-based prevalence surveys in over a dozen countries^7–18^.

One challenge of validating and standardizing serological assays is that they often lack a gold standard control. There are a limited number of standardized controls available for serological assays through the National Institute of Biological Standard Controls, but these often rely on pooled serum, which can be difficult to generate and validate. A renewable reagent would benefit serological assays by omitting the necessity of identifying serum to create positive control pools. A positive control reagent would benefit *C. trachomatis*-serological testing in several ways. The reagent could be used to assess competency of trainees learning any of the Pgp3 antibody assays; it could be used in test development to improve existing tests; and it could be used to standardize across studies by converting semi-quantitative data from MBA or ELISA to concentrations or to validate other cutoff methodologies, such as mixture models^19,20^. Here we present data on the development of a mouse-human chimeric monoclonal antibody to Pgp3 and the validation of immunoassays using this monoclonal antibody.

## Methods

### Generation of monoclonal antibodies (MAb)

#### Development of mouse monoclonal antibodies against recombinant Pgp3 antigen

Protocols were approved by the Institutional Animal Use and Care Committee (IACUC) at the United States Centers for Disease Control and Prevention. All experiments were performed in accordance with relevant guidelines and regulations and reported in compliance with ARRIVE guidelines. Two Balb/c mice and one A/J mouse were included in the study. There were no control groups or randomization as this was not a classical animal study. Sample size was determined based on the predicted number of B-cells needed for fusions and reduction in the number of mice to obtain haplotype diversity in the monoclonal CDRs. Mice were immunized with 50 µg recombinant Pgp3 antigen tagged with glutathione S-transferase (GST) plus adjuvant and boosted on days 14 (50 µg Pgp3 + adjuvant) and 28 (25 µg Pgp3 only). Spleens were harvested for B-cell isolation on day 30. SP2-IL6 myelomas cells (ATCC, Manassas, VA, USA) were fused in 1:1 ratio with isolated splenic B cells^21^. The fused hybridoma cells were plated into 20 mL of semi-solid methylcellulose-based media containing hypoxanthine, aminopterin, and thymidine (STEMCELL, Vancouver, Canada) supplemented with 200 µL of a fluorescein-labeled mouse IgG (Fc) specific CloneDetect (Molecular Devices, San Jose, California, USA) and 10 µL of human interluekin-6 (Roche, Basel, Switzerland). After ten days of plating, clones grown in the presence of CloneDetect were screened by ClonePix 2 system (Molecular Devices, San Jose, California, USA) per the manufacturer’s instructions.

A total of 318 positive clones were selected based on the ranking of fluorescence intensity and then further screened by a fast ELISA for antibodies to Pgp3. Briefly, 100 µL of hybridoma supernatants collected from each selected clone was incubated on plates coated with purified Pgp3-GST recombinant protein and GST protein and detected with a horseradish peroxidase (HRP) conjugated goat anti-mouse IgG at a 1:5000 dilution. Clones were selected for high reactivity to Pgp3 and not GST, resulting in 35 hybridomas chosen for label-free bio-layer interferometry (BLI) analysis with Octet Red96 (Forte Bio, Fremont, CA, USA). Anti-mouse IgG Fc capture sensors were pre-equilibrated for 10 min in 0.01 M PBS, pH 7.2. Microplate wells were filled with 200 µL of hybridoma supernatants and agitated at 1,000 rpm at 30 °C. When all the sensors were saturated with anti-Pgp3 antibodies, purified recombinant Pgp3 antigen was loaded to compare the antibody-antigen affinity rate. Data were analyzed by Octet Data Analysis 9.0 (Forte Bio, Fremont, CA, USA).

The 17 top clones from BLI analysis were further screened by Pgp3 lateral flow assay (LFA)^5^ to analyze reactivity and analytical sensitivity. Clones were tested in five-fold dilutions ranging from 1.28 picograms to 2.5 µg in normal human serum (NHS) for a final volume of 10 µL. Each dilution was added to 50 µL of LFA buffer (1X PBS, 0.5% bovine serum albumin [BSA], 1% Tween 20, 0.02% NaN_3_) in a well of a flat bottom 96-well plate. LFA buffer containing 1 µL of Pgp3-gold and 1 µL of streptavidin (SA)-gold (20 µL total volume) was also added to each well. LFA dipsticks were placed in each well, incubated for 15 min, and scored as positive or negative.

#### Production of human chimeric antibody

The top 5 clones based on BLI, ELISA, and LFA results were selected for hybridoma sequencing. Total RNA for each clone was extracted using an RNeasy Mini kit (Qiagen, Hilden, Germany). Mouse IgG heavy chain and light chain were amplified by Qiagen OneStep RT-PCR kit (Qiagen, Hilden, Germany) and sequenced by GENEWIZ (GENEWIZ, South Plainfield, NJ, USA). To generate humanized chimeric antibodies against Pgp3, the variable domain of heavy chain and light chain from the mouse IgG sequences and constant (Fc) from human IgG were designed, optimized, and synthesized before subcloning into pTT5 vector (GenScript, Piscataway, NJ, USA). After transfection into Chinese hamster ovary (CHO) cells, the human chimeric antibody against Pgp3 was harvested and purified by HiTrap MabSelect SuRe column (GenScript).

These 5 purified chimeric anti-Pgp3 antibodies were tested by LFA, ELISA and MBA. The top 2 chimeric clones, 1C3 and 1G1, were then subcloned into pCDNA3.4 vector respectively for antibody production. 12 days after transfection into ExpiCHO-S cells, the supernatant was harvested and purified by protein G Sepharose 4 fast flow column. The purified human chimeric mAb clones 1C3 and 1G1 were dialyzed in 0.01 M PBS, pH 7.2 and used for further validation.

### General test procedures

#### Lateral flow assay (LFA)

Pgp3-LFA dipsticks were manufactured and samples tested as previously described^22^. Pgp3 was conjugated to 400 nm black latex (Pgp3-latex) using a latex conjugation kit (Abcam, Cambridge, UK) per the kit protocol. Pgp3-latex and SA-gold (Arista Biologicals, Allentown, PA, USA) were diluted 1:120 in PBST (0.3% Tween-20 in PBS) to create a conjugate mastermix. Samples were diluted 1:12 in conjugate mastermix and incubated overnight at 4 °C. The next day, Pgp3 LFA-dipsticks were added to each well and incubated for 20 min. Only valid tests, with a distinct control line, were scored as positive (+) or negative (−) by two readers. If a positive test had a particularly faint test line, this was noted as (+ F).

#### Multiplex bead assay (MBA)

Samples were diluted 1:400 in Buffer B (1X PBS [phosphate buffered saline], 0.5% casein, 0.5% polyvinyl alcohol [PVA], 0.8% polyvinylpyrrolidone [PVP], 0.3% Tween-20, 0.02% sodium azide and 3 µg/mL *E. coli* extract) and tested by MBA as described previously^3^. Briefly, Pgp3-coupled MagPlex^®^ microspheres (Luminex, Austin, TX USA) were incubated with diluted sample for 1.5 h then washed with 0.05% Tween-20 in 1X PBS (PBST). Beads were washed with PBST and incubated with biotinylated mouse anti-human IgG and biotinylated mouse anti-human IgG4 for 45 min to detect anti-Pgp3 antibody bound to beads. After 3 additional washes with PBST, beads were incubated for 30 min with phycoerythrin-labeled streptavidin (SA-PE) to detect bound biotinylated anti-human IgG. After 3 more washes with PBST, loosely bound antibodies were removed with a final 30-min incubation in PBST containing 0.5% BSA and 0.02% sodium azide. Beads were washed one more time, resuspended in 1X PBS, and stored overnight at 4 °C. The next day, plates were read on a Bio-Plex 200 instrument (Bio-Rad, Hercules, CA, USA) equipped with Bio-Plex manager 6.0 software (Bio-Rad). The median fluorescence intensity (MFI) was recorded for each sample.

#### Enzyme-linked immunosorbent assay (ELISA)

Immulon 2HB plates (Thermo Fisher Scientific, Waltham, MA) were prepared as previously described^4^. Plates were coated with 300 ng/mL of Pgp3 antigen in NaCO_3_ pH 9.6 overnight at 4 °C. The next day, wells were washed 4 times with PBST (0.3% Tween-20 in 1X PBS) and incubated for 30 min with 100 µL of StabilCoat^®^ (SurModics, Eden Prairie, MN, USA). Buffer was decanted from wells and plates were dried in a vacuum oven at 30–40 °C for 4 h. Dried plates were stored in foil packages with a 1 g of desiccant at 4 °C until use.

Assays were performed using a double-antigen format with Pgp3-HRP as a detection reagent. Pgp3 was directly conjugated to HRP using Lightning-Link HRP Conjugation Kit (Abcam, Cambridge, UK) as per the kit protocol. Samples were diluted 1:50 in 1X PBS containing 0.3% Tween-20 (PBST2) and tested by ELISA as described previously. Briefly, diluted sample (50 µL) was added to each well of a Pgp3 coated ELISA plate and incubated for 2 h. Wells were washed 4 times with 200 µL of PBST2. Pgp3-HRP was diluted 1:1000 in PBST2 and 50 µL was added to each well to detect any bound anti-Pgp3 immunoglobulin. After a 1-h incubation, plates were washed 4 times in 200 µL of PBST2. TMB (3,3’,5,5’-tetramethylbenzinidine) was added (50 µL/well) and incubated for 30 min. To stop the reaction, 50 µL 1 M H_2_SO_4_ was added per well. The plates were read immediately at 450 nm on a microplate reader and absorbance values were recorded for each sample.

### Validation testing

#### Precision testing

Precision experiments were planned and executed to maximize measurement across testing days and operators following methodology described in Clinical Laboratory and Standards Institute (CLSI) EP05-A3 “Evaluation of Precision of Quantitative Measurement Procedures”.

Clones 1G1 and 1C3 were diluted in NHS to create 12-point curves with twofold dilutions. Sample concentrations ranged from 19.5 to 40,000 ng/mL for the MBA and 2.4 to 5000 ng/mL for the ELISA. Final assay concentrations ranged between 0.05 and 100 ng/mL for both assays. Samples were run in triplicate on 5 different days by 2 operators using the same lot of all reagents on both assays.

Repeatability was determined as the average percent coefficient of variation (%CV) between replicates on the same plate (N = 3 determinations/sample). Within-laboratory precision was determined as the %CV between replicates on all plates for both operators (N = 30 determinations/sample).

#### Limit of blank (LoB)

An initial estimate of the LoB for each clone was calculated using assay blanks (no sample) as a zero-level calibrator for both the MBA and ELISA assays (as per CLSI EP17-A2, Appendix A). Five anonymized normal human sera previously determined to be negative for anti-Pgp3 IgG by MBA were used as blank samples during detection limit testing by both MBA and ELISA. LoBs were calculated using a non-parametric approach for both operator and lots, and the higher value was used as the LoB for the assay (as per CLSI EP17-A2 section 5.3.3.1).

#### Accuracy and limit of detection (LoD)

##### Quantitative assays

Experiments were planned and executed to maximize variability of operators and reagents following methodology described in CLSI EP17-A2 “Evaluation of Detection Capability of Clinical Laboratory measurement”.

Clones 1G1 and 1C3 were diluted in NHS to create 12-point standard curves with twofold dilutions. For the MBA, the sample concentrations for the standard curve ranged from 2 to 4000 ng/mL, and the final concentration (after 1:400 dilution) in the assay ranged from 0.005 to 10 ng/mL. Samples with final assay concentrations of 5, 4 and 0.3125 ng/mL were created and treated as “unknowns” in the MBA. For the ELISA, the sample concentrations for the standard curve ranged from 4.9 to 10,000 ng/mL for the ELISA, and after 1:50 dilution (as per the ELISA protocol), the final assay concentrations ranged from 0.098 to 200 ng/mL for the ELISA. Samples with a final assay concentration of 80, 40 and 20 ng/mL were created and treated as “unknowns” in the ELISA. Low antibody level samples were generated by spiking NHS with monoclonal antibody clones in a range of 1–5 times the LoB. All samples were run in quadruplicate on 3 different days by 2 operators using 2 different lots of reagents.

For interpolation of spiked “unknowns”, average values of standard curve samples by operator for each clone and assay were log transformed and fit with 5 parametric logistic equations in GraphPad Prism 9 (GraphPad Software, CA, USA). Average MFI and absorbance values of unknown samples were converted into ng/mL using the appropriate standard curve for each clone and assay. Accuracy was determined as the calculated concentration / actual concentration × 100%.

LoDs were calculated using a non-parametric approach for each operator and lot and the higher value was used as the LoD for the assay (as per CLSI EP17-A2 section 5.3.3.2). MFI and absorbance values were converted into ng / mL using the appropriate standard curves for each clone and assay by the methods described above.

##### LFA

Clones 1G1 and 1C3 were diluted in NHS to 2.3 to 300 ng/mL for a final assay dilution of 0.2–25 ng/mL. Testing was performed in triplicate by two operators using two lots of LFAs, sample dilutions, and assay reagents. All LFAs were read by two readers for a total of 6 determinations per operator per sample. The LoD was set at the lowest concentration for each clone where 100% of determinations were indicated as positive.

All data reported in results are referring to the concentration of the monoclonal antibody in the sample.

#### Stability testing

##### MBA

1G1 and 1C3 that had been diluted in NHS for precision testing (see above) were aliquoted and stored at − 20 °C. One µL of diluted sera was also spotted onto TropBio filter paper extensions (TropBio Pty Ltd., Townsville, Queensland, Australia), allowed to dry overnight, then stored at − 20 °C. Sera diluted 1:400 in Buffer B (see above) were stored at 4 °C. After 22 months, samples were prepared and tested by MBA following the methods described above, with the exception that for samples stored at 4 °C, the 1:400 dilution that had been stored was added directly to the assay. The correlation coefficient and slope were determined for each clone and storage condition between baseline (day 1 precision testing) and results from the post-storage time point.

##### ELISA

1G1 and 1C3 that had been diluted in NHS for precision testing (see above) were aliquoted and stored at 4 °C and − 20 °C. Four µL of diluted sera was spotted onto TropBio filter paper extensions (TropBio Pty Ltd., Townsville, Queensland, Australia), allowed to dry overnight, then stored at − 20 °C. After 23 months, samples were diluted 1:50 in PBST-milk buffer and tested by ELISA following the methods described above. The correlation coefficient was determined for each clone and storage condition between baseline (day 1 precision testing) and results from the post-storage time point.

##### LFA

Clones 1G1 and 1C3 were diluted in NHS to 37.5 to 1200 ng/mL for a final assay dilution of 18.75 to 100 ng/mL and tested by LFA using the methods described above to generate baseline results. An aliquot of each dilution for each clone was stored for 22 months at 4 °C and − 20 °C. Additionally, 5 µL of diluted sera was spotted onto filter paper, dried, and stored at − 20 °C. After 22 months, samples were prepared and tested by LFA following the methods described above.

Storage conditions and time points for each assay are show in Supplemental Table 1.

**Table 1:** Repeatability and within lab precision coefficient of variation of MBA and ELISA signal measured by two operators.

Conc (ng/mL)	MBA				Conc ng/mL	ELISA			
	1G1		1C3			1G1		1C3	
	%CV_R_	%CV_WL_	%CV_R_	%CV_WL_		%CV_R_	%CV_WL_	%CV_R_	%CV_WL_
40,000	0.7	1.7	0.3	2.5	5000	4.1	12.9	3.2	15.1
20,000	0.4	3.1	0.5	3.2	2500	4.4	14.8	4.5	18.1
10,000	0.6	3.3	0.7	3.7	1250	3.9	16.4	6.0	24.4
5000	1.1	4.8	1.0	5.5	625	3.4	9.5	6.1	20.4
2500	1.8	6.9	1.2	6.0	313	3.6	9.2	5.6	22.0
1250	2.4	1.3	3.4	4.3	156	5.7	9.2	5.7	15.6
625	6.9	10.5	9.7	6.8	78	9.3	12.7	4.7	12.5
313	6.7	7.9	9.0	17.5	39	6.1	10.9	5.6	7.6
156	9.1	13.9	7.1	13.3	20	9.1	13.0	6.5	9.9
78	9.3	7.7	8.8	14.5	10	17.5	48.9	5.6	9.8
39	7.7	8.8	4.6	9.4	5	3.2	27.1	9.9	18.5
20	7.9	8.7	5.4	11.8	2	17.1	53.8	6.9	10.8

Percent CV by sample concentration for each clone is shown is shown for MBA and ELISA for repeatability testing (replicates on the same plate) and within lab precision (multiple plates, multiple operator). %CV_R_ = repeatability coefficient of variation; %CV_WL_ = within-laboratory precision coefficient of variation; MBA = Multiplex bead assay.

## Results

### Screening and characterization of Pgp3 mouse monoclonal antibodies

Out of 318 hybridoma colonies isolated from semi-solid cloning of fusion material, the top 35 clones which reacted to Pgp3 and not the GST tag were selected by ELISA using purified Pgp3-GST and GST (Supplemental Fig. 1A). 17 clones were selected for further characterization based on assay performance by BLI (Supplemental Fig. 1B). Based on both BLI and Pgp3 LFA testing, 5 clones with high analytical sensitivity (testing positive at the low concentrations) were harvested and applied to hybridoma sequencing. The most variable part of complementarity-determining regions (CDR), the CDR3 domain, encoded amino acids that are summarized in Supplemental Table 2. As CDRs are crucial to the diversity of antigen specificities, the CDR3 sequence in Supplemental Table 2 demonstrated different paratopes (i.e. amino acids on the antibody interacting with the amino acids on the epitope of the antigen) for each clone.

**Figure 1:**
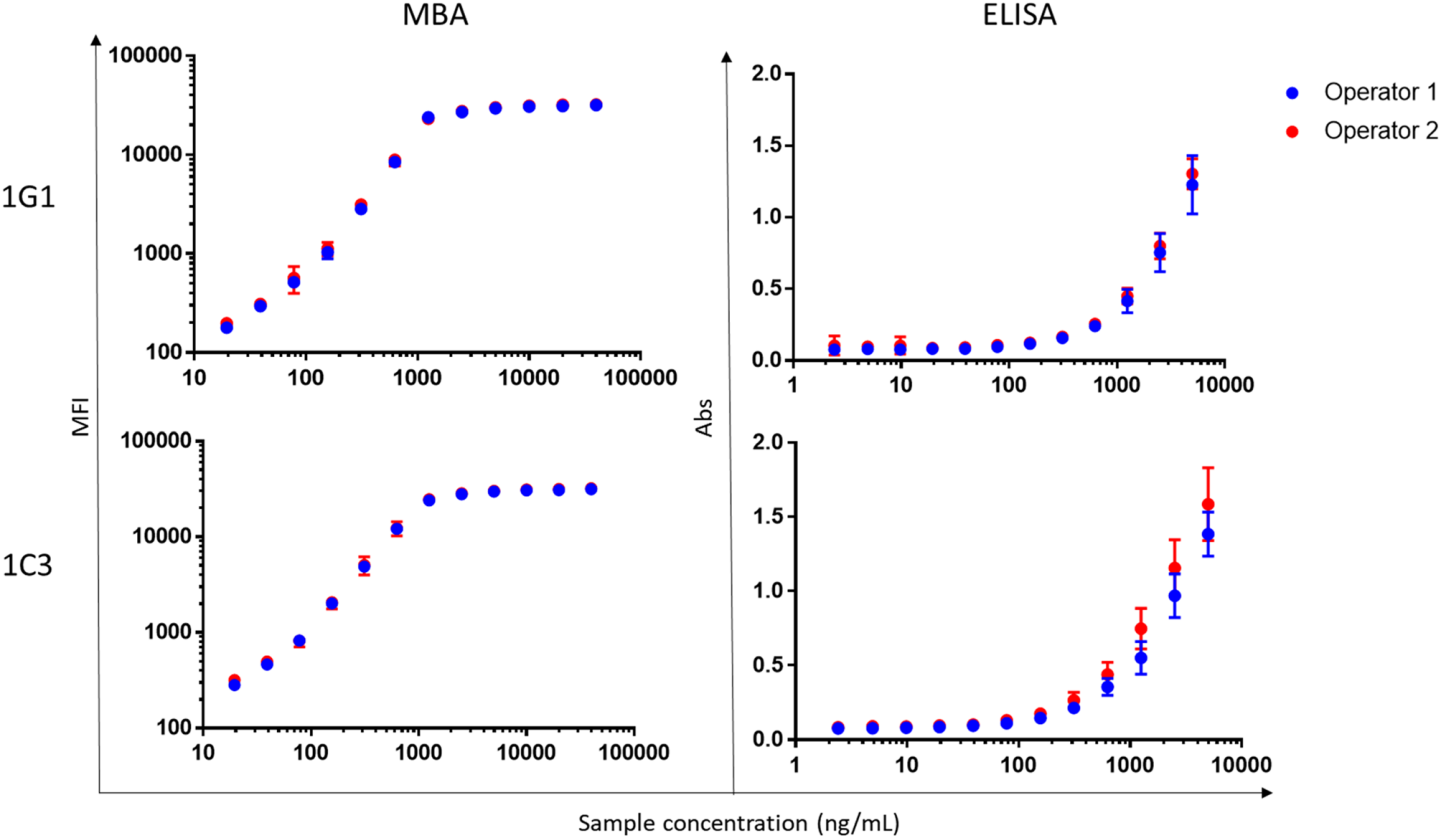
Precision samples for MBA and ELISA by operator. MFI (for MBA testing) and Abs (for ELISA) testing by sample concentration for each clone. Samples tested by operator 1 are in blue and operator 2 are in red. Each point is an average of all replicates tested (15/sample) by each operator. Error bars on each point represent standard deviation. MFI data are presented on a log/log scale to better display the linear range of the assay. MFI = Median fluorescence intensity, Abs = Absorbance, MBA = Multiplex bead assay.

**Table 2:** Accuracy by MBA and ELISA.

Sample	MBA				ELISA			
	Operator 1		Operator 2		Operator 1		Operator 2	
	1G1	1C3	1G1	1C3	1G1	1C3	1G1	1C3
High	120	106	103	113	101	93	99	108
Medium	100	115	87	94	99	88	98	100
Low	108	91	99	99	97	95	96	104

Accuracy by MBA and ELISA for each clone and operator is shown with a high, medium, and low positive sample. Accuracy = calculated concentration/expected concentration × 100. MBA = Multiplex bead assay. High positive sample = 2000 ng/mL by MBA and 4000 ng/mL by ELISA. Medium positive sample = 1600 ng/mL by MBA and 2000 ng/mL by ELISA. Low positive sample = 125 ng/mL by MBA and 1000 ng/mL by ELISA.

### Screening of mouse-human chimeric antibodies for further characterization

Five purified mouse-human chimeric anti-Pgp3 MAbs were tested by MBA at various concentrations in NHS. Clones 1G1 and 1C3 produced the highest MFI values in the assay linear range (from 1000 to 25,000 MFI-bg) (Supplemental Fig. 2). Similar results were seen by LFA and ELISA (data not shown). Based on these data, 1G1 and 1C3 were selected for the full validation by all three assays.

**Figure 2:**
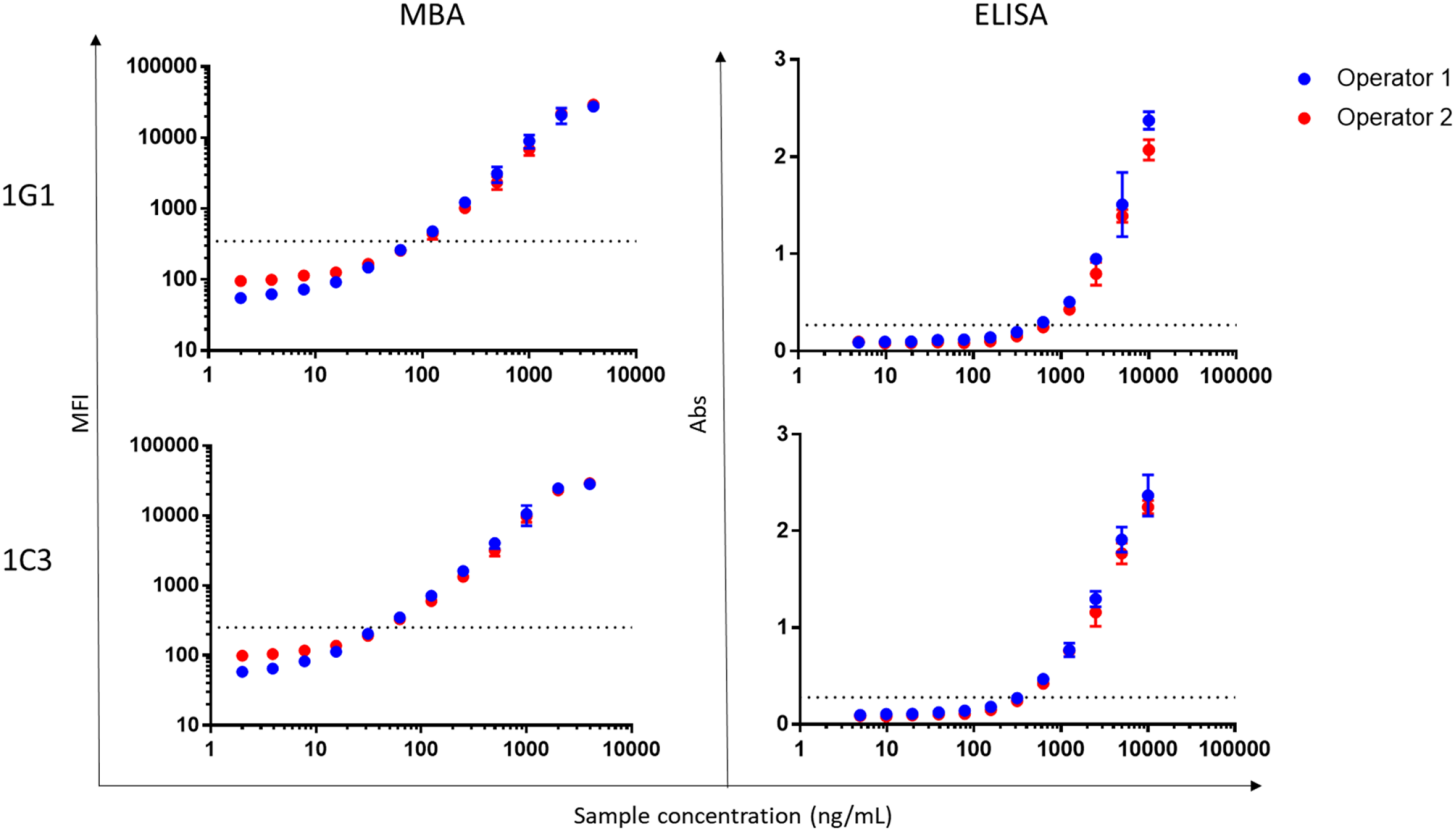
Limit of detection standard curve samples by clone, assay, and operator. MFI (for MBA testing) and Abs (for ELISA) testing by sample concentration (in ng/mL) for each clone. Samples tested by operator 1 are in blue and operator 2 are in red. Each point is an average of all replicates tested (12/sample) by each operator. Error bars on each point represent standard deviation. MFI = Median fluorescence intensity, Abs = Absorbance, MBA = Multiplex bead assay.

### Precision

Repeatability and within-lab precision for MBA and ELISA for each clone are shown in Table 1. Percent CVs were below 20 at all concentrations for repeatability and within-lab precision on MBA and for repeatability on ELISA. Percent CVs for within-lab precision on ELISA tended to be higher than MBA. Values on each assay at each sample concentration by operator are shown in Fig. 1. Dilutional linearity was observed for both assays, with values decreasing proportionally to sample concentration in the assay linear range.

### Accuracy

Accuracy for each clone by operator is shown for high, medium, and low value samples on both MBA and ELISA in Table 2. Almost all (23/24) values were in the acceptable range of between 80 and 120%, while 19/24 had values between 90 and 110% (i.e. were within 10% of the expected value).

### Limits of blank and detection

The limit of blank (LoB) was 236 MFI for MBA. The limit of detection (LoD) of the MBA was 57.6 ng/mL (249 MFI) for 1G1 and 44.8 ng/mL (250 MFI) for 1C3 (Table 3).

**Table 3:** Limit of Detection by Assay.

Assay	1G1		1C3	
	Value	ng/mL	Value	ng/mL
MBA (MFI)	249	57.6	250	44.8
ELISA (Abs)	0.268	700.0	0.278	382.8
LFA	n/a	75	n/a	75

Limit of detection is shown for raw values (MFI or Abs) and sample concentration (in ng/mL) by assay and clone. MBA = multiplex bead assay. LFA = lateral flow assay. MFI = median fluorescence intensity. LFA = lateral flow assay.

The LoB was 0.156 absorbance at 450 nm (OD_450_Abs) for ELISA. The LoD of the ELISA was 700.0 ng/mL (0.268 OD_450_ Abs) for 1G1 and 382.8 ng/mL (0.278 Abs) for 1C3 (Table 3).

The LoD of the LFA was 75 ng/mL for both 1G1 and 1C3 (Table 3). Positive tests were seen starting at a concentration of 18.7 ng/mL (Supplemental Table 3).

Values for standard curve samples on each assay at each sample concentration by operator are shown in Fig. 2.

### Stability testing

For MBA, correlation coefficients were > 0.990 and slopes were between 1 and 1.1 for each clone and storage condition after 22 months of storage (Supplemental Fig. 3). For ELISA, correlation coefficients were > 0.970 for all clones and conditions after 23 months of storage, except for the 1G1 cloned stored at 4 °C (Supplemental Fig. 4). Slopes were between 1 and 1.1 for the 1C3 clone stored at all conditions but ranged between 0.3 and 0.9 for the IG1 clone depending on the storage condition (Supplemental Fig. 4). For LFA, stored specimens tested positive at the same or lower concentrations for all clones and storage conditions (Supplemental Table 4).

## Discussion

Ensuring high quality data for serological studies is critical to generating reliable estimates of population-level exposure and facilitated by the ability to validate and standardize tests. However, validation and standardization of serological tests can be challenging in the absence of gold standards. We present here data on a positive control reagent for serological tests for the human pathogen *Chlamydia trachomatis*. Using this reagent, we show that three different methods for testing for antibodies to Pgp3—a bead-based immunoassay (MBA), ELISA, and rapid test (LFA)—all show excellent, robust, and stable test performance.

There were some quantitative differences between the assays in the parameters evaluated. The precision for the MBA was slightly higher than for ELISA. This may be due to the enzymatic detection used in the ELISA being sensitive to minor changes in temperature, which may not be as consistent between runs as the fluorescence detection of the MBA. A much greater difference in performance between the MBA and ELISA was seen in the limit of detection: the LOD was > 1-logfold lower for MBA than for ELISA. This may be due to the strength of the fluorescence detection in the MBA compared to the light absorption in the ELISA. The MBA uses beads in suspension that may facilitate the antibody binding to antigen on the beads compared with the static nature of antigen capture of antibody in the ELISA. The LOD was slightly lower for LFA than ELISA, which is a bit surprising considering that the ELISA has an amplification step that is lacking in the LFA. Each clone was stable in multiple matrices and temperatures for 22–23 months with the exception of the 1G1 antibody stored diluted 1:400 at 4 °C. Together the data support long-term stability at cold temperatures but suggest that the ELISA is slightly inferior to the MBA and LFA. Therefore, further surveillance efforts should focus on the MBA and LFA.

Beyond the assay performance validation shown here, a positive control anti-Pgp3 chimeric monoclonal antibody has a number of potential use cases. This antibody could be used for validating different lots of each assay, including confirmation of antigen coupling to beads for MBA; standardizing training and competency testing of new staff, performing as assay controls; and normalizing semi-quantitative readouts of ELISA or MBA to standardize data across sites, with the potential to use the antibody to generate standard curves on ELISA or MBA to obtain quantitative data.

There are some limitations to this work. This reagent is only for antibody tests with Pgp3 as the antigenic target. While this is an immunodominant antigen that allows for high throughput serological testing, there is a possibility that antigens with better characteristics may be identified for seroepidemiology of *C. trachomatis,* or that serological tests to evaluate vaccine candidates other than Pgp3 will be needed. In those instances, these data can provide proof-of-concept for developing *C. trachomatis*-specific reagents or could be used to validate a serum panel that would have applicability to a larger number of antigens than this reagent. The reagent also needs further reproducibility testing in outside laboratories.

Test validation can cover a wide variety of parameters, but these can broadly be categorized as test performance and fit-for-purpose. The data presented here address test performance parameters such as accuracy and repeatability. Other studies have assessed the sensitivity and specificity of these tests to detect ocular *C. trachomatis* infection in children and found 90–95% sensitivity (i.e. 90–95% of children with a PCR-positive ocular swab also test positive for antibody) and 96–99% specificity, using non-endemic pediatric specimens^3,11,23^. For trachoma, use cases include post-validation surveillance and providing data on transmission in unmapped areas or supporting clinical and infection data to provide a fuller picture of transmission^1,24^. For urogenital chlamydia, proposed use cases for serological tests are measuring infection, measuring disease, and for use in vaccine studies^2,25^. Having standardized reagents to support these tests will increase confidence in the data obtained to support public health initiatives.

## Supplementary Information

Supplementary Information.

## Data Availability

The datasets used and/or analyzed during the current study is available from the corresponding author on reasonable request.
