# Supplementary material for: Validation of immunoassays for the Chlamydia trachomatis antigen Pgp3 using a chimeric monoclonal antibody
Source: Sci Rep. 2023 May 4;13:7281. doi: 10.1038/s41598-023-33834-4 (PMC10160048; doi:10.1038/s41598-023-33834-4)
Supplement: Supplementary file 1 — Supplementary Information. [file 41598_2023_33834_MOESM1_ESM.docx]

| **Assay** | **Time point** | **4°C storage** | **-20°C storage** |
| --- | --- | --- | --- |
| **MBA** | 22 months | Stored as sera diluted 1:400 in Buffer B | Stored as neat sera and dried onto filter paper |
| **ELISA** | 23 months | Stored as neat sera | Stored as neat sera and dried onto filter paper |
| **LFA** | 22 months | Stored as neat sera | Stored as neat sera and dried onto filter paper |

**Supplemental Table 1**. Storage conditions for monoclonal antibodies used in stability testing for each of the assays.

 
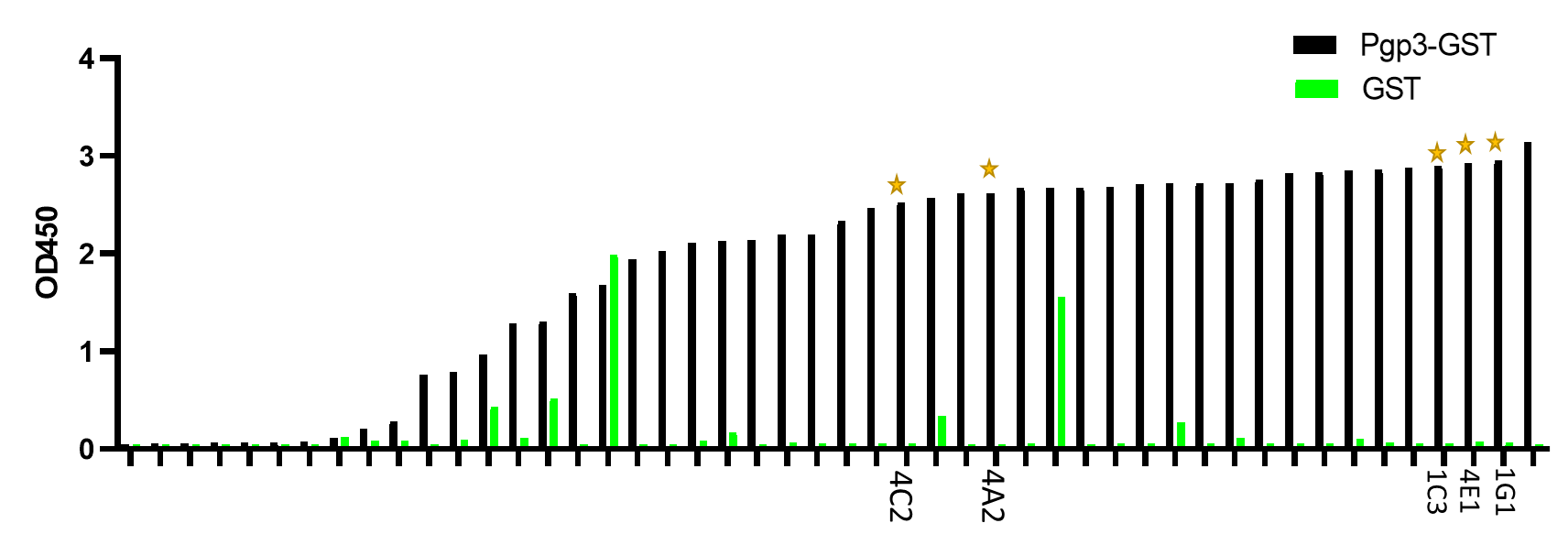


**Supplemental Figure 1 A. ELISA screening of anti-Pgp3 mouse mAbs.** Supernatants from clones were screened for antibodies to Pgp3 (black bars) and the fusion protein GST (green bars). Stars represent the clones selected for chimerization; these are labeled on the x-axis with the clone ID. OD450- optical density at 450 nm; GST- glutathione S-transferase.


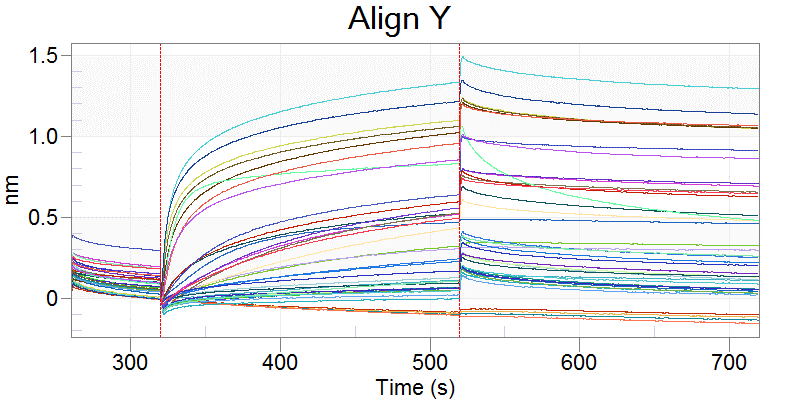


Association

Disassociation

PBS baseline

4A2

4A2

4C2

4C2

4E1

4E1

1G1

1G1

1C3

1C3

**Supplemental Figure 1 B. Biolayer interferometry (BLI) screening of anti-Pgp3 mouse mAbs.** Association and dissociation curves are shown; clones selected for chimerization are labeled.

| **Clones** | **CDR3 in Heavy chain** | **CDR3 in Light (Kappa) chain** |
| --- | --- | --- |
| **4C2** | CTNFDGYYRAWFAYW | CQQHYSTPFTF |
| **4A2** | CARGGYYYDNTYYAMDSW | CQHHYGIPWTF |
| **1C3** | CARGLRGYAMDYW | CQQYSSFPPTF |
| **4E1** | CARLGPLLYYFDYW | CQQYNNYPLTF |
| **1G1** | CARSTSYYMDYW | CQQYNYYPPTF |

**Supplemental Table 2. CDR3 Sequencing for 5 clones of pgp3 mAbs.** The amino acid sequences of the complementarity determining region 3 (CDR3) for the heavy and light chains of the five clones selected for chimerization are shown.

**Supplemental Figure 2: Functional screening of mouse-human chimeric monoclonal antibody clones by multiplex bead assay.** MFI-bg for 5 humanized chimeric antibody clones at various sample concentrations in ng/mL. Each clone is represented by a different color. MFI-bg = Median fluorescence intensity minus background (assay buffer alone).

|  | **1G1** | | **1C3** | |
| --- | --- | --- | --- | --- |
| **ng/mL** | **Operator 1** | **Operator 2** | **Operator 1** | **Operator 2** |
| **300** | 100% | 100% | 100% | 100% |
| **150** | 100% | 100% | 100% | 100% |
| **75** | 100% | 100% | 100% | 100% |
| **38** | 83% | 100% | 67% | 83% |
| **19** | 0% | 17% | 17% | 0% |
| **9.4** | 0% | 0% | 0% | 0% |
| **4.7** | 0% | 0% | 0% | 0% |
| **2.3** | 0% | 0% | 0% | 0% |

**Supplemental Table 3: LFA Limit of Detection Testing.** The percent of samples testing LFA positive (6 replicates / operator) is shown for each clone and operator by sample concentration (in ng/mL).

**
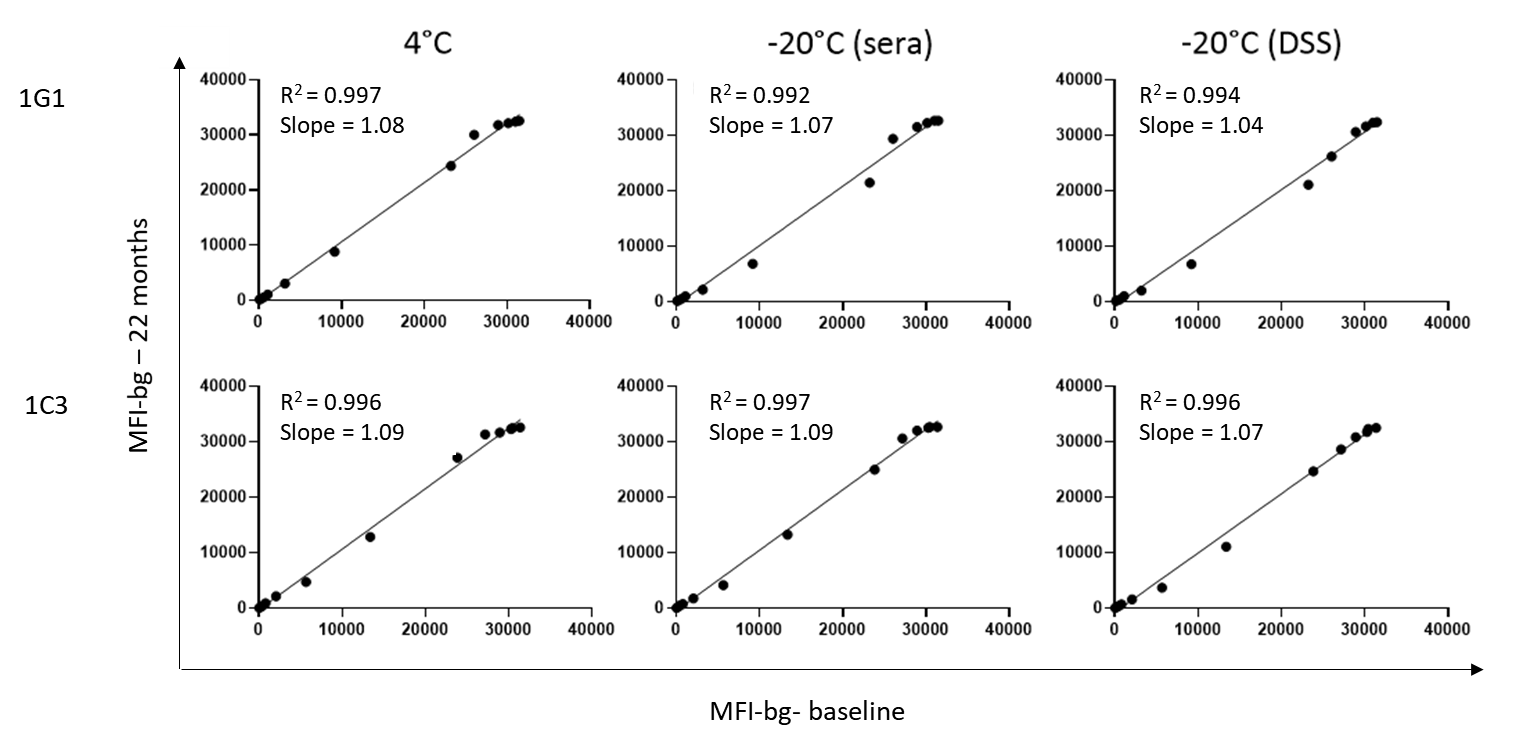
**

**Supplemental Figure 3. Stability of Pgp3 chimeric monoclonal antibody clones 1G1 and 1C3 at cold temperatures in a multiplex bead assay (MBA).** MFI-bg at baseline testing and after 22 months of storage is shown at various concentrations for each clone at three different storage conditions. Sera was diluted 1:400 in Buffer B and stored at 4°C (“4°C”), stored at -20°C (“-20°C sera”), and dried onto filer paper and stored at -20°C (“-20°C DSS). MFI-bg = median fluorescence intensity minus background, Buffer B = 1X PBS [phosphate buffered saline], 0.5% casein, 0.5% polyvinyl alcohol [PVA], 0.8% polyvinylpyrrolidone [PVP], 0.3% Tween-20, 0.02% sodium azide and 3 µg/mL *E. coli* extract **,** DSS = dried serum spots.

**
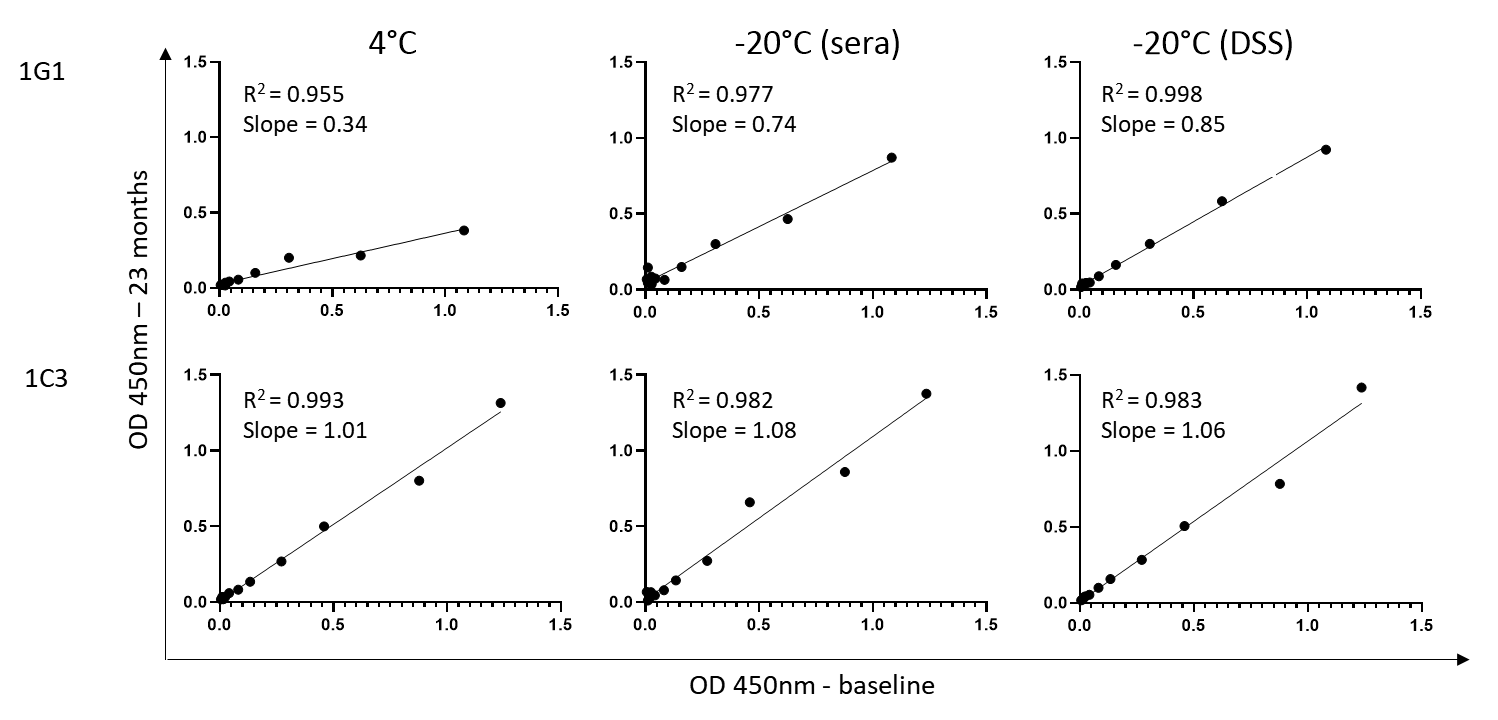
**

**Supplemental Figure 4. Stability of Pgp3 chimeric monoclonal antibody clones 1G1 and 1C3 at cold temperatures in an ELISA.** OD 450 at baseline testing and after 23 months of storage is shown at various concentrations for each clone at three different storage conditions. Sera was aliquoted and stored at 4°C (“4°C”) and -20°C (“-20°C sera”). Sera was also dried onto filer paper and stored at -20°C (“-20°C DSS). OD 450 = optical density at 450 nm, DSS =dried serum spot, ELISA = Enzyme-linked immunosorbent assay.

|  | **ng/mL** | **Baseline** | **4°C**  **liquid** | **-20°C liquid** | **-20°C spots** |
| --- | --- | --- | --- | --- | --- |
| **1G1** | **1200** | + | + | + | + |
|  | **600.0** | + | + | + | + |
|  | **300.0** | +F | +F | +F | +F |
|  | **150.0** | - | +F | +F | +F |
|  | **75.0** | - | - | - | - |
|  | **37.5** | - | - | - | - |
| **1C3** | **1200** | + | + | + | + |
|  | **600.0** | + | + | + | + |
|  | **300.0** | + | +F | + | + |
|  | **150.0** | +F | +F | +F | +F |
|  | **75.0** | - | - | +F | +F |
|  | **37.5** | - | - | - | - |

**Supplemental Table 4: Stability of each clone at various storage conditions on LFA.** LFA results baseline testing and after 23 months of storage is shown at various concentrations for each clone at three different storage conditions. Sera was aliquoted and stored at 4°C (“4°C”) and -20°C (“-20°C sera”). Sera was also dried onto filer paper and stored at -20°C (“-20°C spots). Samples testing positive are indicated by “+” and highlighted in dark green. Samples testing positive with faint test lines are indicated as “+F” and highlighted in light green.
